# Seroprevalence of anti-hepatitis E virus antibodies in domestic pigs in Mexico

**DOI:** 10.1186/s12917-017-1208-z

**Published:** 2017-09-21

**Authors:** Montserrat Elemi García-Hernández, Mayra Cruz-Rivera, José Iván Sánchez-Betancourt, Oscar Rico-Chávez, Arely Vergara-Castañeda, María E. Trujillo, Rosa Elena Sarmiento-Silva

**Affiliations:** 1 Facultad de Medicina Veterinaria y Zootecnia, Universidad Nacional Autónoma de México, Ciudad de México, 04510 México; 2 Facultad de Medicina, Universidad Nacional Autónoma de México, 04510 Ciudad de México, México; 3 Facultad de Ciencias Químicas, Universidad La Salle, Benjamín Franklin 47, 06140 Ciudad de México, México

**Keywords:** HEV, Mexico, Pigs, Seroprevalence

## Abstract

**Background:**

Hepatitis E virus (HEV) infection is one of the most common causes of acute liver diseases in humans worldwide. In developing countries, HEV is commonly associated with waterborne outbreaks. Conversely, in industrialized countries, HEV infection is often associated with travel to endemic regions or ingestion of contaminated animal products. Limited information on both, human and animal HEV infection in Mexico is available. As a consequence, the distribution of the virus in the country is largely unknown. Here, we assessed the seroprevalence of HEV among swine in different geographical regions in Mexico.

**Methods:**

Seroprevalence of anti-HEV antibodies in swine herds in Mexico was evaluated in a representative sample including 945 pig serum specimens from different regions of the country using a commercial enzyme-linked immunosorbent assay (ELISA).

**Results:**

The overall prevalence of anti-HEV antibodies in swine was 59.4%. The northern region of Mexico exhibited the highest seroprevalence in the country (86.6%), while the central and southern regions in Mexico showed lower seroprevalence, 42.7% and 51.5%, respectively.

**Conclusions:**

In Mexico, HEV seroprevalence in swine is high. Importantly, northern Mexico showed the highest seroprevalence in the country. Thus, further studies are required to identify the risk factors contributing to HEV transmission among pigs in the country. Assessment of HEV human infection in the context of viral transmission in swine is required to better understand the epidemiology of hepatitis E in Mexico.

## Background

Hepatitis E virus (HEV) infection is an emerging disease of increasing importance. HEV affects approximately ∼20 million persons annually worldwide, causing ∼70,000 deaths. In humans, HEV is transmitted primarily by the fecal-oral route [1, 2]. Clinically, hepatitis E is indistinguishable from other viral hepatitis. Hepatitis E is usually a self-limiting disease in immunocompetent individuals, commonly resulting in mild symptoms or asymptomatic disease [3–6]. Occasionally, and for reasons not completely understood, HEV infection can progress to fulminant hepatitis [7]. The overall mortality rate of HEV infection ranges from 0.5 to 4%; however, it is considerably higher among pregnant women (∼20%) [2].

HEV is a non-enveloped virus with a single-stranded positive-sense RNA genome of ca. 7.2 (kb) in length. HEV is a member of the family *Hepeviridae* within the genus *Orthohepevirus*. This genus comprises four species: *Orthohepevirus A*, *Orthohepevirus B, Orthohepevirus C* and *Orthohepevirus D* –all grouping viruses that infect birds and different mammals. Based on its genetic variability, *Orthohepevirus A* is classified into seven main genotypes (HEV1–7). Genotypes 1 and 2 infect exclusively humans while all other are considered zoonotic. Genotypes 3 and 4 also affect pigs, wild boars, rabbits and mongoose. Genotype 5 and 6 have been identified from wild boar, while genotype 7 infects camels.

HEV is endemic or epidemic to Africa and Asia. Epidemics of HEV in endemic regions are usually associated with water-borne outbreaks [1, 8]. Individuals from non-endemic regions who acquire HEV infection often have a history of traveling to endemic regions and/or consumption of contaminated animal products [9] evidence suggests that autochthonous HEV infections in developed countries also occur [7, 10]. HEV has been reported to circulate in different countries in Latin America, including Mexico [11]. After the first reported HEV outbreak in Mexico in 1987 [12], when HEV genotype 2 was originally described, several research groups have subsequently reported the circulation of HEV in the country in both, swine and humans [2, 13, 14]. In Mexico, HEV surveillance is not routinely performed; and as a result, diagnosis of HEV-related disease is underreported. Likewise, monitoring of HEV infection in pigs is rare [15]. Thus resulting in a profound lack of information about human and animal infection.

In pigs, reduced feed intake and mild diarrhea may be observed, but evident clinical disease signs such as elevation of liver enzymes or bilirubin levels are usually not detected. Upon infection, experimentally HEV-inoculated pigs seroconvert to anti-HEV immunoglobulin IgG, shedding of virus in feces is observed during approximately 23 days in contact infected pigs [16, 17]. Importantly, identification of HEV RNA in liver tissue and bile is not uncommon [18]. Higher anti-HEV antibody prevalence among individuals in close contact with pigs (handlers), in comparison to normal population has been previously reported [19]. In these settings, HEV-related disease has been related to consumption of contaminated meat products.

In this study we aimed to assess the seroprevalence of HEV in domesticated pigs in different geographical regions in Mexico.

## Methods

### Serum samples

**Fig. 1:**
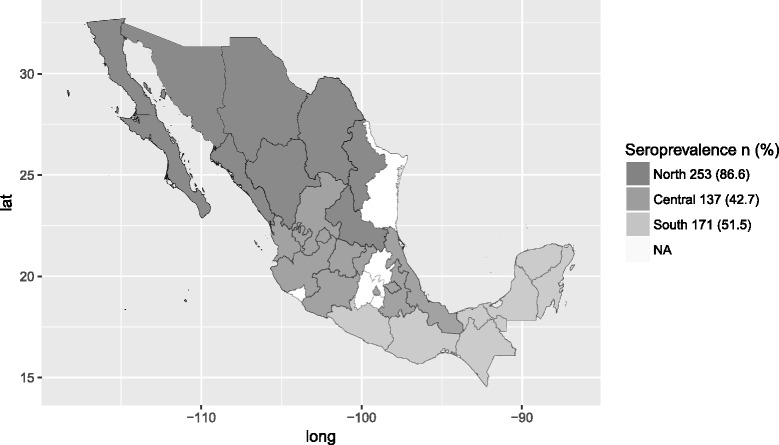
Map showing the percentage of anti-HEV positive pigs and classification by region of the states included in the study. The central region included samples from Ciudad de Mexico, Guanajuato, Jalisco, Michoacán, Nayarit, Puebla, Queretaro, Aguascalientes, Tlaxcala, Veracruz and Zacatecas states, south from Quintana Roo, Campeche, Chiapas, Guerrero, Oaxaca, Tabasco and Yucatan; and northern states Baja California, Chihuahua, Coahuila, Durango, Nuevo Leon, Sinaloa, San Luis Potosi and Sonora. Latitude (Long). Latitude (lat). NA (Not available)

Serum samples were obtained from different farms in 2014 and 2015 with informed consent from the owners. A total of 945 representative swine serum samples were selected for this study. Both, industrialized farms (95%) and backyard herds (5%) located in different counties from 27 states in Mexico were included. Samples were grouped according to their collection site and divided into three geographical regions (central, southern and northern Mexico) (Fig. 1). The sample size for statistical significant result for each region was calculated using the Daniel algorithm [20].

### Detection of anti-HEV antibodies

Detection of anti-HEV antibodies was performed using a commercial enzyme-linked immunosorbent assay (ELISA) (Wantai Biopharmaceutical, Inc. Beijing, China), using horseradish peroxidase-labeled protein A (Bio-Rad, CA, USA) as reporting conjugate. Briefly, serum samples were diluted (1:10), and incubated for 30 min at 37 °C. After washing, the conjugate was added and incubated for 30 min at 37 °C. Plates were washed, and 100 μl of substrate solution (tetramethylbenzidine, Wantai Biopharmaceutical) were added. The reaction was stopped after 15 min with 50 μl of stop solution. The absorbance was measured at 450 nm using multi-scan EX spectrophotometer (Thermo Fisher, Waltham, MA). The cut off value was calculated after blanking as the mean absorbance of the negative controls +0.16 (mean absorbance value for negative controls +0.16) according to the manufacturer’s instructions. Serum specimens exhibiting an absorbance value greater than the cut-off value were considered positive for anti-HEV antibodies.

### Statistical analyses

Statistical analyses were performed using the Statistical Package for the Social Sciences (IBM SPSS Statistics v22.0, USA). All reported *p*-values were two-sided and a p-value of less than 0.05 was deemed statistically significant. Prevalence of positive serum among swine categories and regions were assessed by the chi- squared test and odds ratios were determined to evaluate the risk of positive/negative results for qualitative results, according to location and production stage.

In order to know the effect of each state in the corresponding region in the hepatitis seroprevalence we performed a general linear model (glm) with binomial distribution. The positive or negative result of hepatitis seroprevalence was used as dependent variable, while the region, state within region and production stage within region were used as independent variables. These analysis were performed selected the states with at least 10 observations, after the selection the sample size was equal to 878. A likelihood test was performed to determine the overall significance of the logistic model. The odd ratio of each state and region was calculated by exponentiating the model estimates. An analysis of variance was also calculated to know the statistically difference in hepatitis seroprevalence by state.

## Results

A total of 945 samples were analyzed, 292 (30.8%) specimens were collected from the northern region of Mexico, and 332 sera from the southern (35.1%) and 321 central parts of Mexico (34%) and, when classified according to the production stage, a total of 299 samples corresponded to weaned production, 323 to fattening and 323 to reproducers animals (Table 1). No differences in distribution for this factor were found among the total of the samples.

**Table 1:** Classification of analyzed samples by region and production stage

Category	Total	North	South	Central
Weaned	299 (31.6)	88 (29.4)	115 (38.4)	96 (32.1)
Fattening	323 (34.2)	94 (29.1)	116 (35.9)	113 (35)
Reproducers	323 (34.2)	110 (34)	101 (31.3)	112 (34.7)
		292 (30.8)	332 (35.1)	321 (34)

Data shown as n (%)

The overall prevalence of anti-HEV IgG in the country was 59.4%. The highest seroprevalence was found in northern Mexico (86.6%), compared to 42.7 and 51.5% in the central and southern regions, respectively (*p* < 0.001) (Fig. 1). Based on the production stage, the chi- squared test showed no significant differences in positivity between the different production stages in the northern and central regions; however, in southern Mexico, a significant difference was observed in fattening swine compared to weaned and reproducer animals (*p* = 0.012) .

In the general linear model (glm) with binomial distribution, only the states with at least 10 observations were selected (*n* = 878), in this analysis, the production stage within region didn’t show a statistical significance (LR chisq = 9.755, DF = 6, *p* = 0.135) (AIC = 948.84) while the state within region showed a statistical significance (LR chisq = 115.56, DF = 13 *p* < 0.01) (AIC = 946.6). The central region was used as intercept in the glm (Table 2). No significant difference was found in the analysis of variance in hepatitis seroprevalence by state (DF = 2, F = 2.59, *p* = 0.118).

**Table 2:** Out put of general lineal model. The region and the state within region were used as independent variables. Only states with >10 observations were considered

Region	State	Estimate	Std. Error	z value	P	N	Prevalence (%)	OR	2.50%	97.50%
Center (intercept)		0.588	0.558	1.054	0.292	299	44.1	1		
	DF	−0.728	0.636	−1.144	0.253	43	46.5	0.483	0.13	1.637
	Guanajuato	−0.007	0.599	−0.011	0.991	92	64.1	0.993	0.285	3.127
	Jalisco	−1.314	0.6	−2.188	0.029*	92	32.6	0.269	0.077	0.847
	Michoacan	−2.821	0.825	−3.421	0.001*	31	9.7	0.06	0.01	0.271
	Queretaro	−0.588	0.843	−0.697	0.486	10	50.0	0.556	0.101	2.905
	Veracruz	−1.194	0.754	−1.583	0.113	17	35.3	0.303	0.064	1.282
North		2.105	0.627	3.356	0.001*	256	89.5	8.205	2.258	27.636
	Nuevo Leon	−1.083	0.618	−1.752	0.08	24	83.3	0.339	0.108	1.288
	Sinaloa	−2.162	0.491	−4.404	0.000*	27	63.0	0.115	0.044	0.305
South		1.284	0.774	1.658	0.097	323	52.0	3.611	0.794	17.653
	Chiapas	−3.163	0.636	−4.974	0.000*	51	21.6	0.042	0.011	0.134
	Guerrero	−1.664	0.654	−2.544	0.011*	29	55.2	0.189	0.047	0.638
	Oaxaca	−0.599	0.687	−0.872	0.383	32	78.1	0.549	0.13	2.051
	Tabasco	−4.27	1.174	−3.635	0.000*	12	8.3	0.014	0.001	0.098
	Yucatan	−1.765	0.559	−3.159	0.002*	169	52.7	0.171	0.049	0.463

Odds ratio (OR)

GLM

*p < 0.05

## Discussion

Here, we have shown a high prevalence of HEV-specific antibodies in domesticated pigs in different geographical regions of Mexico. The results suggested that animals in intensive pig farms are in contact with HEV at an early production stage. Few studies have reported the circulation of HEV in Mexico, particularly in pigs [15]. As a consequence, limited information about the seroprevalence of HEV in swine is available. A recent study, conducted in nine states located in central Mexico showed a seroprevalence of 30% [15]. The discrepancies between the seroprevalence found between the studies can be attributed to the difference in geographical areas tested and the methodology used by Merino-Ramos and cols which consisted of an ELISA test based on the use of recombinant HEV 3 ORF-2 expressed in *Trichoplusia ni* larvae as antigen. Performances of anti-HEV antibody detection methods vary significantly [21]. Here, we used a well-known commercial assay for the detection of antibodies in order to minimize false antibody results [22–28]. Interestingly, HEV seroprevalence in pig herds in Mexico has been reported as high as 81% [2]. Both previous studies were focused in particular geographical regions in Mexico. Here, we undertook a nation-wide approach to assess the HEV seroprevalence in swine in the country, showing that northern Mexico is the most affected region by HEV infection in pigs. Nonetheless, high seroprevalence in all three geographical regions was observed. The reasons for these differences in seropositivity among different parts of Mexico could be associated with common practices in pig farms such as high population density, and shorter production cycles that significantly increased the risk for HEV infection [16].

Studies aimed to detect the virus in acutely infected animals are necessary to identify the routes of transmission exploited by the virus to warrant persistence in the pig population.

## Conclusions

The relatively high seropositivity observed in all regions in Mexico suggests abundant circulation of HEV and high transmission rates among pigs. These findings highlight the importance of this zoonosis in Mexico. Further nation-wide studies on HEV prevalence in human population are required to better understand the epidemiology of HEV in Mexico. Assessing the prevalence of HEV infection in high-risk populations such as pig handlers and immunocompromised patients is critical to estimate HEV burden in the country. Implementation of adequate HEV surveillance in Mexico is important to implement proper control measures aimed to prevent virus spread.

## Abbreviations

CI: Confidence interval
ELISA: Competitive enzyme-linked immunosorbent assay
HEV: Hepatitis E virus
OR: Odds ratio
